# Identification and validation of lactylation-related genes signature and immune infiltration landscape of rheumatoid arthritis based on machine learning

**DOI:** 10.1186/s41065-025-00579-1

**Published:** 2026-05-14

**Authors:** Xiaoli Hu, Qian Xiao, Lizhou Wang, Yuan Xu, Qiaoqiao Gou, Jing Wen, Shi Zhou

**Affiliations:** 1 Ultrasound Center, Affiliated Hospital of Guizhou Medical University, No. 28 Guiyi Street, Guiyang, 550001 People’s Republic of China; 2 Ultrasound Center, Guiyang Public Health Clinical Center, Guiyang, People’s Republic of China; 3 Department of Interventional Radiology, Affiliated Hospital of Guizhou Medical University, No. 28 Guiyi Street, Guiyang, 550001 People’s Republic of China

**Keywords:** Rheumatoid arthritis, Lactylation-related genes, Immune infiltration landscape, Diagnostic biomarker, Machine learnings

## Abstract

**Background:**

The pathogenic mechanisms underlying rheumatoid arthritis (RA) remain elusive. Lactylation, a novel post-translational modification, may regulate immune and metabolic reprogramming, underscoring the imperative to delineate lactylation-related genes (LRGs) driving RA progression.

**Methods:**

LRG expression profiles from RA patients and healthy controls were analyzed from GEO datasets. Immune infiltration and LRG-immune correlations were assessed. A machine-learning framework identified a hub LRG signature, whose metabolic and therapeutic relevance was evaluated via functional enrichment and druggability analyses. qRT-PCR validated hub gene expression in RA MH7A cells model.

**Results:**

Transcriptomic profiling identified 36 differentially expressed LRGs regulating cytokine networks and immune signaling in RA. Disease stratification revealed two molecular subtypes, with Subtype B demonstrating p53 signaling and innate immunity pathway activation via gene set variation analysis (GSVA). Weighted correlation network analysis (WGCNA) identified subtype B-associated modules (504 genes). A machine learning-derived 7-LRG signature (*Sdc1*, *Pfkfb1*, *Fut8*, *Adh1b*, *Kif23*, *Adh1c*, *Pkc1*) differentiated RA from controls (AUC 0.92). Single-cell resolution analysis localized Sdc1 to plasma cell clusters, correlating with memory B cell expansion and macrophage polarization. Hub LRGs were enriched in glucose metabolism pathways in RA, and *Sdc1*, *Adh1b*, and *Adh1c* were druggable, suggesting potential therapeutic targets. qRT-PCR validation confirmed significant LRG upregulation in RA cellular models.

**Conclusion:**

Our findings establish lactylation as a key modulator of immune dysregulation in RA pathogenesis. Seven LRG biomarkers were identified and validated, exhibiting dual potential as prognostic indicators and therapeutic targets through lactylation-driven pathway modulation for RA.

**Supplementary Information:**

The online version contains supplementary material available at 10.1186/s41065-025-00579-1.

## Introduction

Rheumatoid arthritis (RA) is characterized by an autoimmune response that results in synovitis, ultimately causing potential joint deformities [1]. RA is generally known to be related to environmental and genetic factors, and its mechanisms of action include misdirected immune system attack on the joints, persistent immune cell activation that keeps the joints in a state of chronic inflammation [2]. Finally, the clinical manifestations of the disease are dominated by bone erosion and cartilage [3]. The clinical diagnosis of RA relies on the physical manifestations, symptoms and clinical biomarkers presented by the patient [4]. The identification of distinct biomarkers in RA remains critical for early diagnosis and optimized therapeutic strategies, given the disease’s clinical heterogeneity and current pharmacological limitations despite recent therapeutic advancements.

Lactate, the terminal product of glycolysis, is recognized for its involvement in diverse signaling cascades, exerting regulatory functions in both homeostatic and pathophysiological tissue contexts [5]. Protein lactylation modification is one of pivotal mechanisms through which lactate exerts its regulatory influence on macrophage polarization and associated cellular functions [6], thereby affecting the immune reaction of diseases or tumors. The histone lactylation process is notably upregulated during the terminal phase of M1 macrophage polarization, culminating in the acquisition of an M2 phenotype characterized by anti-inflammatory properties [7]. This transition is instrumental in modulating inflammatory responses, attenuating macrophage activation, and exerting regulatory effects on disease progression. Moreover, recent research has indicated that lactylation plays a significant part in the progression of autoimmune diseases, including systemic lupus erythematosus (SLE) [8], Type 1 diabetes [9] and RA [10]. Fibroblast-like synoviocytes (FLS) are significantly involved in the swelling and bone damage associated with RA [10]. Antimalarial drug artemisinin (ART) has demonstrated a substantial inhibitory impact on the proliferation of FLS in pathological conditions by specifically targeting the lactylation of PKM2, thereby presenting a promising pathway for the development of pharmaceutical interventions of RA [11]. Despite the existence of research on the regulatory function of lactylation in RA, the precise mechanism by which lactylation-related genes (LRGs) modulate immunity in RA and consequently the progression of the disease remains unclear.

Our objective was to discover the possible impact of lactylation in RA and provide a theoretical framework and guidance for the advancement of innovative RA clinical treatments. Various bioinformatic methodologies were employed in this study to assess the role of LRGs in the progression of RA. Seven LRGs have been identified and confirmed as diagnostic biomarkers for RA. Furthermore, an assessment was conducted on the relationship between the diagnostic biomarkers and the pattern of immune cell infiltration landscape in both the normal and RA groups. Our investigation represents an effort in delineating the LRGs signature, which may be utilized as a prognostic indicator and is proposed as a candidate for therapeutic intervention in RA.

## Methods

### Data source

The RNA-sequencing dataset GSE89408 (joint synovial biopsies from 28 normal and 152 RA patients) was downloaded from the GEO database (http://www.ncbi.nlm.nih.gov/geo/). Three microarray datasets GSE12021 (mRNA expression profiles from 9 normal and 12 RA patients), GSE55235 (mRNA expression profiles from 10 normal and 10 RA patients) and GSE55457 (mRNA expression profiles from 10 normal and 13 RA patients) were downloaded from the GEO and merged into one microarray dataset (total 29 normal and 35 RA patients). The batch effect between different datasets was calibrated through the “combat” algorithm in the sva R package.

### Identified the differential expression of LRGs

The LRGs were collated from the molecular signatures database (MSiDB, https://www.gsea-msigdb.org/gsea/msigdb). The differentially expressed genes (DEGs) from the GSE89408 were derived by the R package “DEseq2” between the RA patients and normal (|FC| = 1.5, P.adj.value < 0.05), and the DEGs from the merged microarray were collected through the R package “Limma” (|FC| = 1.5, P.adj.value < 0.05). Then, the correlation between the datasets and the LRGs was evaluated in further analysis. The R package “Corrplot” was applied to demonstrate the correlation between DEGs in RA patient examples.

### Correlation analysis between LRGs and immune characteristics

Based on the single sample gene-set enrichment analysis (ssGSEA), using the CIBERSORT algorithm to analysis the differential infiltration abundance of immune cells between RA and normal, which was visualized by heatmap. The boxplots illustrated the state of immune cell infiltration and differentially expressed LRGs between RA examples and normals.

### Consensus clustering analysis of differentially expressed LRGs

Based on the LRGs, the R package “ConsensusClusterPlus” was performed to consensus clustering analysis of 152 RA samples in GSE89408 and categorize the RA patients into different subtypes. The R package “Rtsne” was applied to show the tSNE plots of subtypes sample distribution. Gene-set variation analysis (GSVA) was performed to identify the salient regulated pathways among the LRGs related subtypes. Heatmap was used to describe the differential enriching pathways between different subtypes. The CIBERSORT algorithm was used to analyze the differences of immune cell infiltration between the two LRGs-related subtypes. Furthermore, the differential LRGs were analyzed between the two LRGs-related subtypes. In addition, consensus clustering analysis was also carried out in merged microarray based on differentially expressed LRGs.

### Identification of co-expression module genes

The DEGs between the two subtypes were subjected to Weighted gene co-expression network analysis (WGCNA) to identify the cluster-related co-expression modules. The samples that were situated in the clusters and exceeded the specified cutoff thresholds were incorporated in the subsequent analysis. Following this, the adjacency matrix was generated and subsequently converted into a topological overlap matrix (TOM) by applying the soft thresholding technique with the power parameter β. Based on the TOM-based dissimilarity metric, genes were categorized into distinct gene modules through the dynamic tree cut algorithm, and modules with highly correlated eigengenes were amalgamated. Lastly, the co-expressed genes were pinpointed through an assessment of the module membership (MM) and gene significance (GS) scores for the genes contained within the designated modules. Furthermore, Kyoto encyclopedia of genes and genomes (KEGG) was conducted to determine the significant pathways involved in the module black gene associated with LRGs cluster B.

### Identification of lactylation‑related hub genes in RA

To screen the LRGs-related hub genes, we conducted the feature selection based on the differentially expressed LRGs. Least Absolute Shrinkage and Selection Operator (LASSO), Random Forest Recursive Feature Elimination (RF-REF) and Support Vector Machine (SVM) were utilized to screen the LRGs that have the ability to distinguish between different subtypes. ROC curve was used to calculate the AUC of hub genes, thereby to evaluate the accuracy and specificity of prediction. The evaluation was performed in GSE89408 and the merged microarray dataset.

### Correlation with hub genes and immune cells infiltration abundance

A correlation study was executed to examine the relationship between key genes and the density of immune cells infiltration in RA samples. To delve deeper into the expression patterns of key genes across various cells, we queried the expression of hub genes through the online analysis website [12].

### Construction of RA cell model

The human rheumatoid arthritis fibroblast synovial cell line: MH7A was acquired from Guizhou Medical University. MH7A cells were cultured in dulbecco’s modified eagle medium (DMEM, Corning, 10-013-CVRC) supplemented with 10% fetal bovine serum (FBS, Gibco, 10099141 C), 100 mg/ml streptomycin/penicillin (Sangon, E607011), under a humidified atmosphere of 5% CO_2_ at 37 °C. Cells were seeded into 6-well plates and serum-starved overnight in DMEM devoid of FBS. Subsequently, cells were exposed to 1 µg/mL lipopolysaccharide (Solarbio, L8880) for 3 h to induce the construction of RA model.

### Quantitative real-time PCR (qRT-PCR)

RNA extraction was performed using the RNA extraction kit (Sigma, T9424) following the manufacturer’s protocol. Subsequently, total RNA was reversed transcribed into cDNA using the RevertAid RT kit (Thermo, K1622). The mRNA expression levels were normalized to β-actin mRNA levels and quantified using the 2^−ΔΔCT^ method. The primers used in this study are listed in Additional file 1. The qRT-PCR results were visualized as bar graphs using GraphPad PRISM software, version 10.1.2.

### Functional enrichment analysis

Gene ontology (GO, https://www.geneontology.org/) and Kyoto Encyclopedia of Genes and Genomes (KEGG, https://www.genome.jp/kegg/) enrichment analyses were conducted to explore signaling pathways associated with the seven hub LRGs.

### Drug-gene interaction and druggability analysis

To assess the therapeutic potential of the identified hub LRGs, we queried the Drug-Gene Interaction Database (DGIdb, https://www.dgidb.org/, version 5.0). The hub genes were input into DGIdb, and matched results were retrieved. Intersecting genes were visualized using Venn diagrams, while drug-gene associations were displayed as interaction networks.

### Statistical analysis

The data processing and collation post-download were conducted using Perl software (Version 5.18.2) in the present study. Data analysis was carried out using R version 3.6.1. *p*-value < 0.05 was deemed statistically significant unless otherwise specified.

## Results

### A total of 36 differentially expressed LRGs were identified in RA

**Fig. 1:**
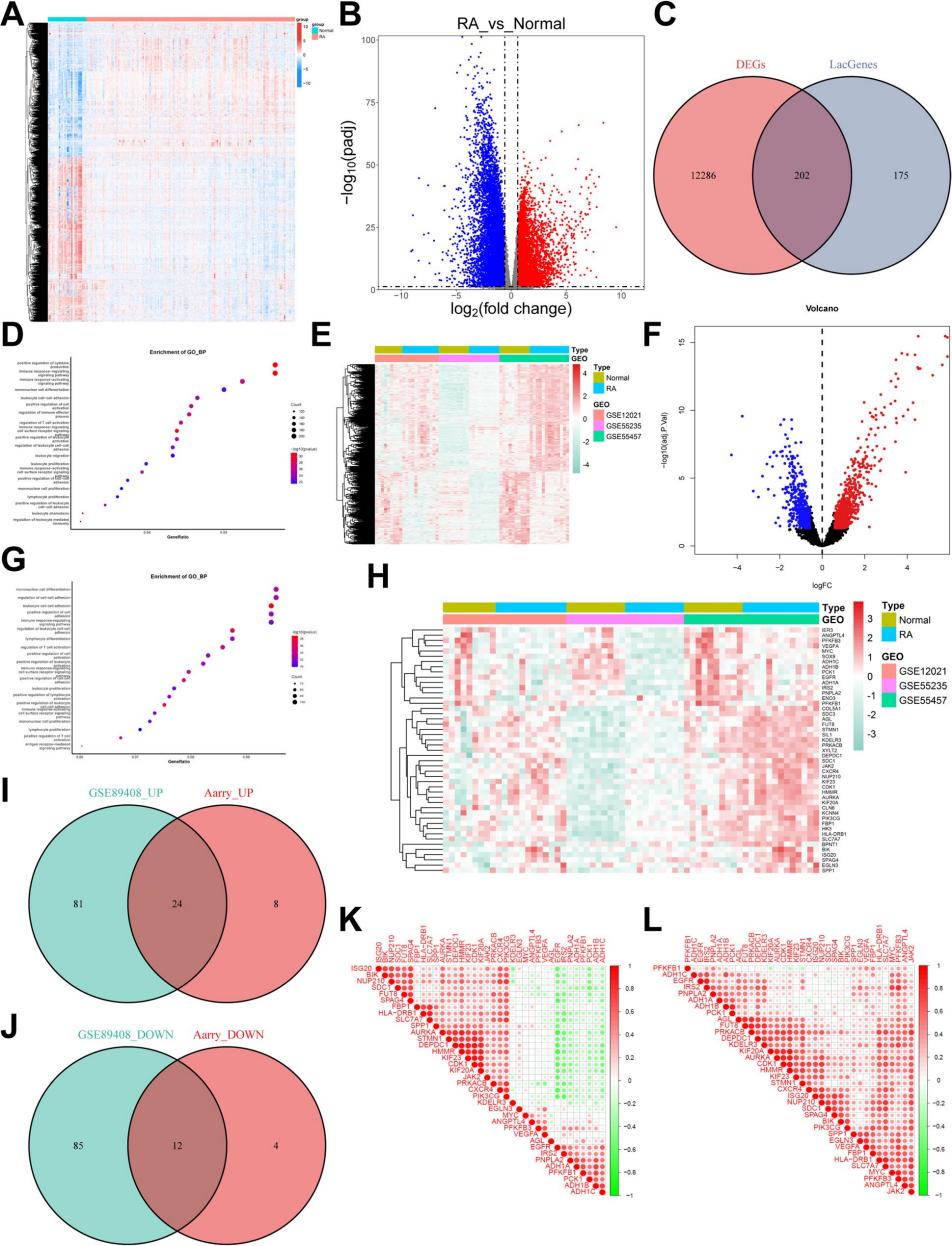
A total of 36 differentially expressed LRGs were identified in RA. (**A**) Heatmap of gene expression levels between the RA and normal group in GSE89408. (**B**) The volcano plot showing the DEGs between the RA and normal group in GSE89408. Red dot indicated notably upregulated genes and blue dot indicated notably downregulated genes. (**C**) A total of 202 differentially expressed LRGs were identified between GSE89408 and LRGs. (**D**) GO enrichment analysis of differentially expressed LRGs in GSE89408. (**E**) Heatmap of DEGs between the RA and normal group in merged microarray. (**F**) The volcano plot showing the DEGs between the RA and normal group in in merged microarray. Red dot indicated notably upregulated genes and blue dot indicated notably downregulated genes. (**G**) GO enrichment analysis of differentially expressed LRGs in merged microarray. (**H**) Heatmap of differentially expressed LRGs between the RA and normal group in merged microarray. (**I**) The Venn plot showing overlapping lactylation-associated upregulated genes GSE89408 and merged microarray. (**J**) The Venn plot shows overlapping lactylation-associated downregulated genes GSE89408 and merged microarray. (**K**) Correlation analysis of upregulated LRGs between GSE89408 and merged microarray in RA. (**L**) Correlation analysis of downregulated LRGs between GSE89408 and merged microarray in RA

The comprehensive research approach was illustrated in Figure S1. Transcriptomic analysis of GSE89408 synovial biopsies identified 12,488 DEGs (7,085 down/5,403 up) in RA patients (Fig. 1A-B). An intersection analysis of MSigDB-derived 376 LRGs with DEGs revealed 202 overlapping genes (Fig. 1C), constituting the analytical matrix. GO enrichment of these LRGs highlighted cytokine regulation and immune signaling pathways as predominant functional clusters (Fig. 1D). The expression mode of LRGs was also identified in merged microarray. Figure 1E showed the expression of DEGs between RA and normal in merged microarray, and volcano plot illustrated the visual representation of DEGs between RA and normal in merged microarray (Fig. 1F). Unlike in GSE89408, the results of GO functional analysis showed that mononuclear cell differentiation, regulation of cell-cell adhesion and leukocyte cell-cell adhesion were prominently associated with LRGs in merged microarray (Fig. 1G). Figure 1H demonstrated the differential expression of LRGs between RA and normal in merged microarray. To further obtain key LRGs, we integrated RNA-seq with differentially expressed LRGs from microarrays. There was a total of 24 overlapping genes that were upregulated both in GSE89408 and the merged microarray (Fig. 1I) and a total of 12 overlapping genes that were downregulated both in GSE89408 and the merged microarray (Fig. 1J). Figure 1K-L illustrated the expression correlation in RA among 24 upregulated and 12 downregulated LRGs. Overall, a total of 36 differentially expressed LRGs in RA were identified both in GSE89408 and the merged microarray, including 24 upregulated and 12 downregulated LRGs.

### Marked infiltration of inflammatory immune cells occurred in RA

**Fig. 2:**
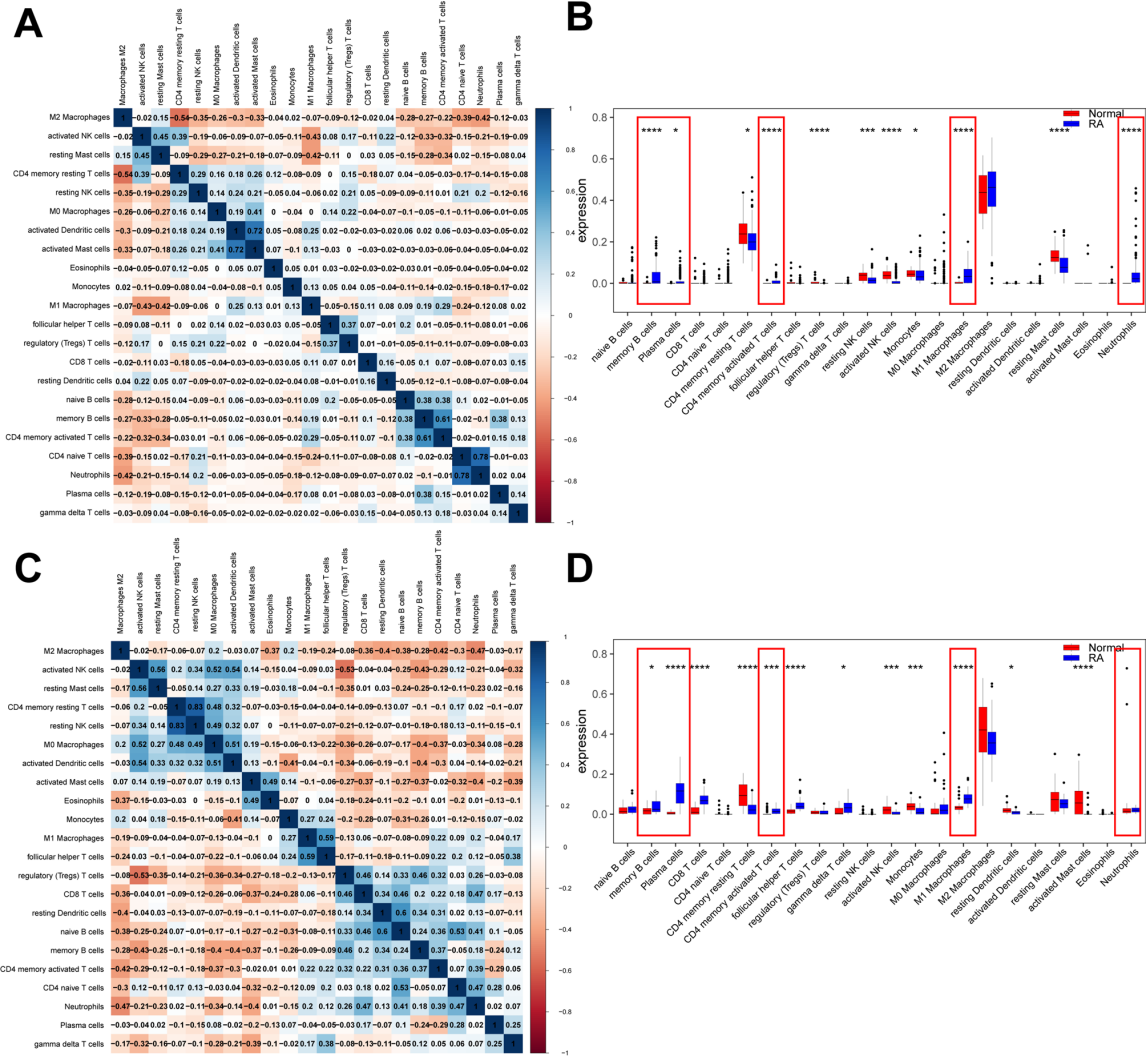
Marked infiltration of inflammatory immune cells occurred in RA. (**A**) Heatmap of differentially expressed immune cells between the RA and normal group in GSE89408. (**B**) Variations in the presence of immune cells between the RA and normal group were examined in GSE89408. (**C**) Heatmap of differentially expressed immune cells between the RA and normal group in merged microarray. (**D**) Variations in the presence of immune cells between the RA and normal group were examined in merged microarray

To explore the variations in the abundance of immune cell infiltration between RA and normal conditions, we performed the CIBERSORT algorithm for assessing the immune cell composition in GSE89408 and the merged microarray. In GSE89408, the abundance of immune cells such as memory B cells, plasma cells, CD4 memory activated T cells, M1 macrophage and neutrophils was more pronounced in RA samples compared to normal samples (Fig. 2A-B), and the same result occurred in the merged microarray (Fig. 2C-D). Therefore, the marked infiltration of inflammatory immune cells occurred in RA.

### LRGs divided RA into two subtypes in GSE89408

**Fig. 3:**
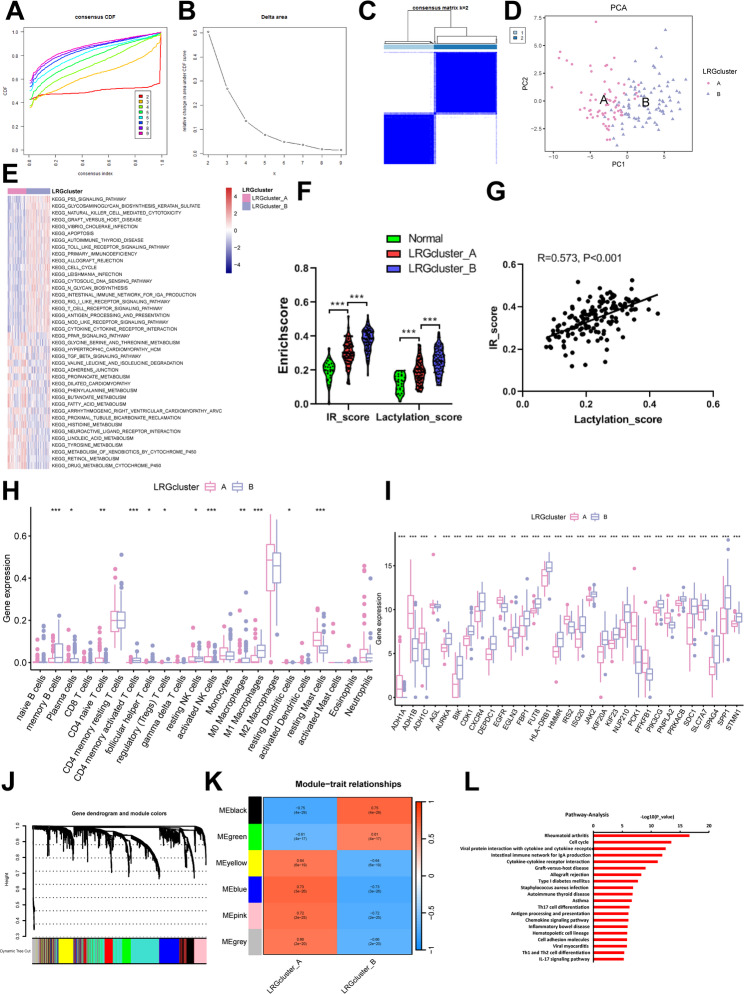
LRGs divided RA into two subtypes in GSE89408. (**A**) The panel displays consensus cumulative distribution function curves for different clusters in GSE89408. (**B**) The delta area plot presents the change in the area under the CDF curve for different numbers of clusters in GSE89408. (**C**) The heatmap visualizes the consensus matrix for κ = 2 clusters in GSE89408. (**D**) The PCA plot illustrates the separation of samples into two main clusters. (**E**) Heatmap of differentially enriched pathways between two subtypes in GSE89408. (**F**) The violin plot shows the level of IR-score and Lactylation-score for each individual sample. (**G**) The scatter plot reveals a strong positive association between the IR-score and Lactylation-score. (**H**) The box plot compares the infiltration levels of various immune cells between two clusters in GSE89408. (**I**) The box plot reveals the expression levels of LRGs between two clusters in GSE89408. (**J**) The gene dendrogram shows hierarchical clustering of modules based on expression profiles. (**K**) The heatmap illustrates the correlation between six modules and two LRGs-related clusters. (**L**) The bar chart displays significantly enriched pathways associated with black module

To investigate the lactylation modification patterns in RA, unsupervised consensus clustering analysis was conducted for RA samples based on expression of 36 distinctly expressed LRGs in GSE89408. The clustering results indicated that selecting κ = 2 appeared to be a favorable choice, pointing out that the lactylation modification profiles were precisely categorized into two distinct subtypes (Fig. 3A-C). A total of 152 RA samples were significantly divided into subtype A and subtype B as visualized in tSNE space (Fig. 3D). These results indicated that LRGs can effectively discriminate RA into two distinct subtypes. In addition, two different subtypes were also identified for the lactylation modification profiles in merged microarray (Figure S2A-C). GSVA enrichment analysis demonstrates that fully activated immune pathways were remarkable enriched in subtype B compared to subtype A, such as: P53 signaling pathway, natural killer cell mediated cytotoxicity, toll like receptor signaling pathway and primary immunodeficiency (Fig. 3E). The ssGSEA algorithm was utilized to determine the inflammatory response-score (IR-score) and Lactylation-score for each individual sample. Results revealed that both the IR-score and Lactylation-score in LRG cluster B were significantly elevated compared to those in LRG cluster A and the Normal group (Fig. 3F). Furthermore, a strong positive association was noted between the IR-score and Lactylation-score (Fig. 3G). To further delve the association between LRGs subtypes and immune characteristics in RA, the CIBERSORT method was implemented to evaluate the disparities in immune cell distribution across the two identified clusters. The box plot showed that B memory cells, CD4 naïve T cells, CD4 memory activated T cells, M0 macrophage and pro-inflammatory M1 macrophage were more infiltrated in subtype B (Fig. 3H). A sum of 22 LRGs were more upregulated in subtype B than subtype A, including AURKA, BIK, CDK1, CXCR4, DEPDC1, EGLN3, FBP1, FUT8, HLA-DRB8, HMMR, ISG20, JAK2, KIF20A, KIF23, NUP210, PIK3CG, PRCAKB, SDC1, SLC7A7, SPAG4, SPP1 and STMN1 (Fig. 3I). Similar results were obtained in merged microarray (Figure S2D). We used the DEGs between the two subtypes for WGCNA in the GSE89408 to analysis the correlation between subtypes and co-expression module genes. Based on the WGCNA, we calculated the correlation between modules and subtypes in RA. The black modules containing 504 genes had extremely positive correlation with subtype B (*r* = 0.75) (Fig. 3J-K). KEGG pathway analysis identified enriched immune pathways in module black (Fig. 3L), including RA progression, cytokine-receptor networks, and IgA-associated intestinal immunity. These findings stratify RA into mechanistically distinct subtypes, with subtype B exhibiting pronounced immune pathway predominance, particularly in cytokine-mediated signaling.

### The hub genes display a strong predictive performance in RA

**Fig. 4:**
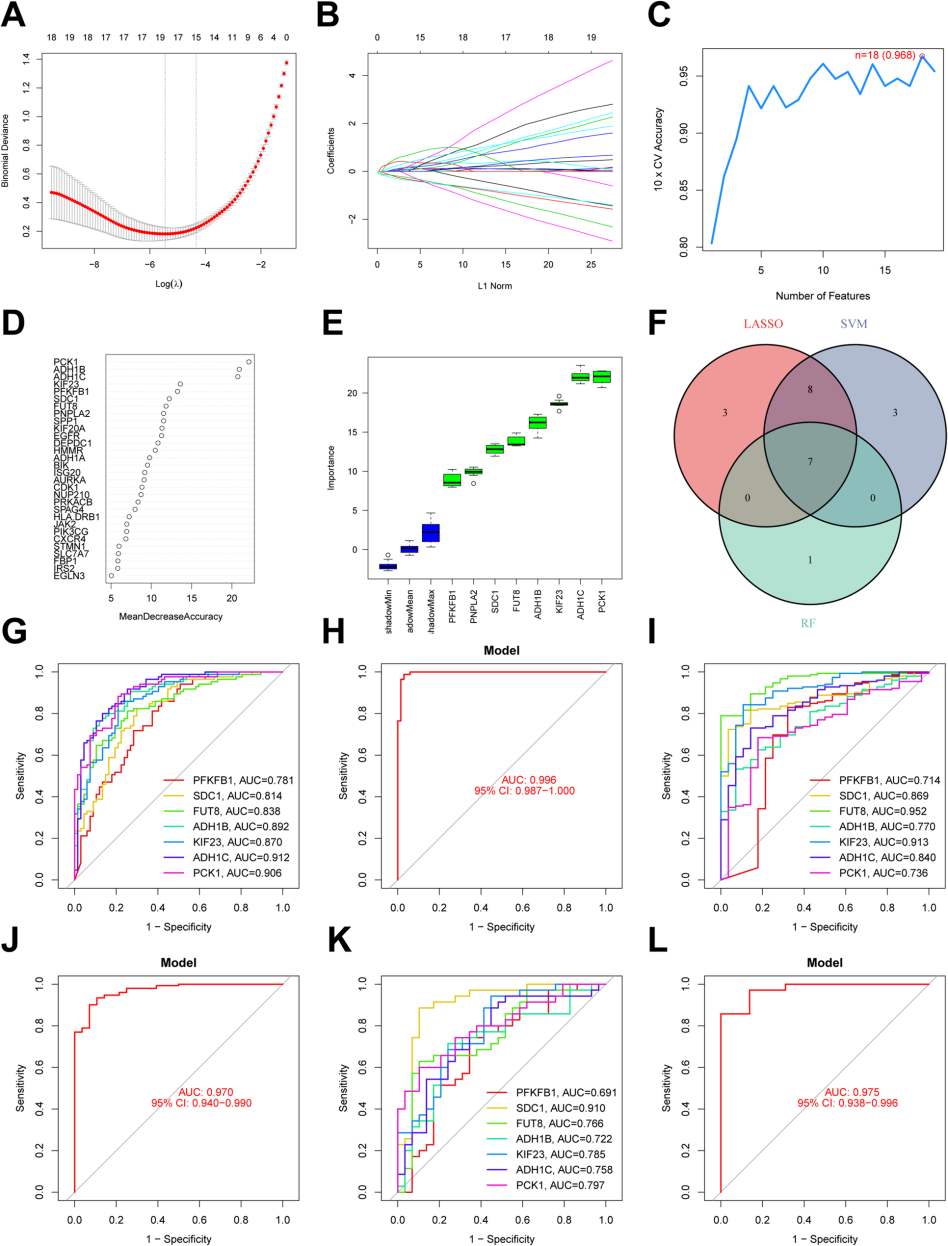
The hub genes display a strong predictive performance in RA. (**A**) The panel illustrates the relationship between binomial deviance and the logarithm of the regularization parameter. (**B**) A total of 18 hub genes were identified by LASSO. (**C**) A total of 18 hub genes were identified by SVM. (**D**) The bar plot indicates the importance of various genes. (**E**) A total of eight hub genes were identified by RF-REF. (**F**) Seven overlapping genes were identified between LASSO, SVM and RF-REF. (**G**) ROC curves showing the robust diagnostics of seven hub genes between two clusters. (**H**) ROC curve demonstrates the strong predictive performance of the LRGs-related diagnostic model between two clusters. (**I**) ROC curves show the robust diagnostic capability of the seven hub genes between the RA and normal group in GSE89408. (**J**) ROC curve demonstrates the strong predictive performance of the LRGs-related diagnostic model between the RA and normal group in GSE89408. (**K**) ROC curves reveal the robust diagnosis ability of seven hub genes between the RA and normal group in merged microarray. (**L**) ROC curve demonstrats the strong predictive performance of LRGs-related diagnostic model between the RA and normal group in merged microarray

To construct an LRG diagnostic signature model to predict RA subtypes, we performed the LASSO, SVM and RF-REF to screen the hub genes. A subset of 18 genes was pinpointed utilizing the LASSO method (Fig. 4A-B), a separate group of 18 genes was discovered through the application of the SVM (Fig. 4C), and a distinct set of eight genes was recognized by RF-REF approach (Fig. 4D-E). Ultimately, we identified 7 overlapping genes (*Sdc1*, *Pfkfb1*, *Fut8*, *Adh1b*, *Adh1c*, *Kif23* and *Pck1*) that may accurately distinguish between two subtypes (Fig. 4F). In addition, we utilized the ROC curves to predict the accuracy and specificity of hub genes and validated the accuracy of the predictive model in GSE89408. Of that, the AUC values of *Pfkfb1* (AUC = 0.781), *Sdc1* (AUC = 0.814), *Fut8* (AUC = 0.838), *Adh1b* (AUC = 0.892), *Kif23* (AUC = 0.870), *Adh1c* (AUC = 0.912) and *Pck1* (AUC = 0.906) were all higher than 0.75, and the predictive model had good accuracy (AUC = 0.996) for the diagnosis of subtypes (Fig. 4G-H). We tried to use hub genes for the diagnosis of RA and found that *Pfkfb1* (AUC = 0.714), *Sdc1* (AUC = 0.869), *Fut8* (AUC = 0.952), *Adh1b* (AUC = 0.770), *Kif23* (AUC = 0.913), *Adh1c* (AUC = 0.840) and *Pck1* (AUC = 0.736) had a better diagnostic effect between RA and Normal in GSE89408 (Fig. 4I-J). The result is the same as in the merged microarray (Fig. 4K-L). This suggests that *Sdc1*, *Pfkfb1*, *Fut8*, *Adh1b*, *Adh1c*, *Kif23* and *Pck1* and the LRG diagnostic signature model could effectively distinguish RA into two subtypes and has good diagnostic value for RA.

### The hub gene Sdc1 was significantly correlated with inflammatory immune cell abundance in RA

**Fig. 5:**
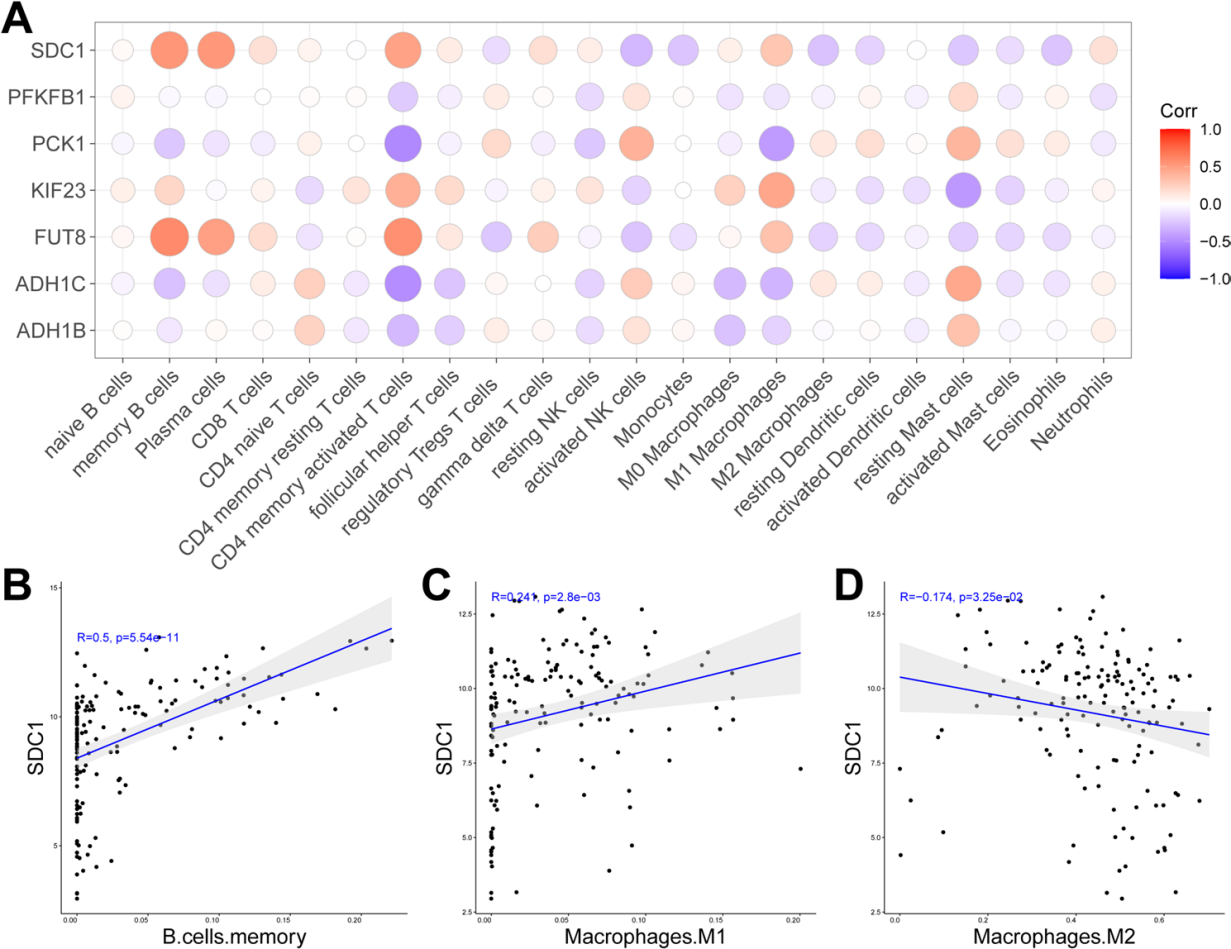
The hub gene *Sdc1* was significantly correlated with the abundance of inflammatory immune cells. (**A**) Association analysis of seven key genes with immune cell proliferation. (**B**) Correlation between hub genes *Sdc1* and the abundance of B cells. (**C**) Correlation between hub genes *Sdc1* and the abundance of M1 macrophages. (**D**) Correlation between hub genes *Sdc1* and the abundance of M2 macrophages

To unravel the linkage between key genes and various immune cells, an examination was conducted to assess the relationships between seven pivotal genes and the prevalence of immune cells within the system. Figure 5A provided a detailed illustration of the relationships between key genes and the abundance of immune cells. The expression level of *Sdc1* demonstrated a very strong positive association with the infiltration abundance of B cells memory and macrophages M1, as well as a significant negative correlation with the infiltration abundance of macrophages M2 (Fig. 5B-D).

**Fig. 6:**
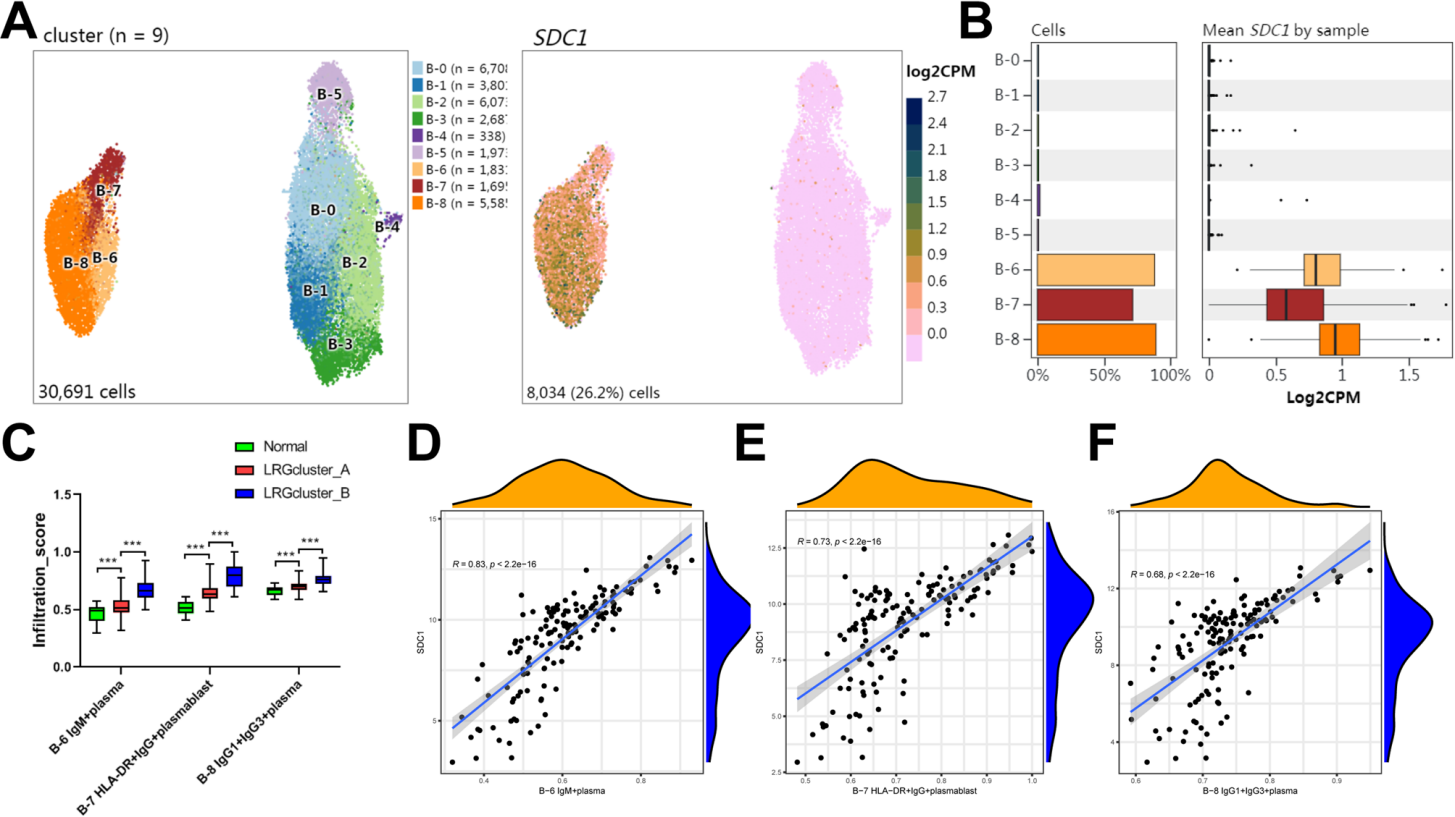
The expressed distribution of *Sdc1* is mainly associated with B cell plasma cells. (**A**) UMAP visualizes the expressed distribution of *Sdc1* in B cells. (**B**) The bar plot shows the expressed distribution of *Sdc1* in B cells. (**C**) The violin plot shows the differences in B cells Infiltration-score between LRG cluster A, LRG cluster B and the normal. (**D-F**) The scatter plot shows the relationship between *Sdc1* and IgG1^+^IgG3^+^ plasma (B-8), IgM^+^ plasma (B-6) and HLA-DR^+^IgG^+^ plasmablast (B-7) clusters of B cells

We further analyzed the expression distribution of hub genes in various immune cells. The results showed that *Sdc1* was mainly distributed in plasma cell clusters of B cells including IgG1^+^IgG3^+^ plasma (B-8), IgM^+^ plasma (B-6) and HLA-DR^+^IgG^+^ plasmablast (B-7) (Fig. 6A-B). *Fut8* was also mainly distributed in plasma cell clusters of B cells including IgG1^+^IgG3^+^ plasma (B-8), IgM^+^ plasma (B-6) and HLA-DR^+^IgG^+^ plasmablast (F-3) (Figure S3A-B). In addition, *Adh1b* was mainly distributed in sublining cell clusters of Stromal cells including CD34^+^ sublining (F-2) and NOTCH3^+^ sublining (F-7) (Figure S3C-D). *Kif23* was mainly distributed in Tph cell or proliferating clusters of T cells including : CD4^+^ Tfh/Tph (T-3), CD4^+^ Tph (T-7) and Proliferating (T-18) (Figure S3E-F). *Adh1c* was mainly distributed in sublining cell clusters of Stromal cells including CD34^+^ sublining (F-2), NOTCH3^+^ sublining (F-7) (Figure S3G-H). *Pkc1* was hardly expressed in immune cells (Figure S3I-J), while none of the expression distribution of *Pfkfb1* in immune cells was significant (Figure S3K-L). Moreover, clusters of the IgG1^+^IgG3^+^ plasma (B-8), IgM^+^ plasma (B-6) and HLA-DR^+^IgG^+^ plasmablast (B-7) had higher infiltration abundance in LRG cluster B compared to LRG cluster A and the normal (Fig. 6C). The scatter plot elucidated that the expression of *Sdc1* was highly positively correlated with the infiltration abundance of three subtypes of B cells [IgG1^+^IgG3^+^ plasma (B-8), IgM^+^ plasma (B-6) and HLA-DR^+^IgG^+^ plasmablast (B-7)] (Fig. 6D-F). Consequently, hub genes were significantly correlated with inflammatory immune cell abundance in RA, and the expressed distribution of *Sdc1* was mainly associated with plasma cells of B cells.

### Hub LRGs are linked to metabolic reprogramming and exhibit potential draggability

**Fig. 7:**
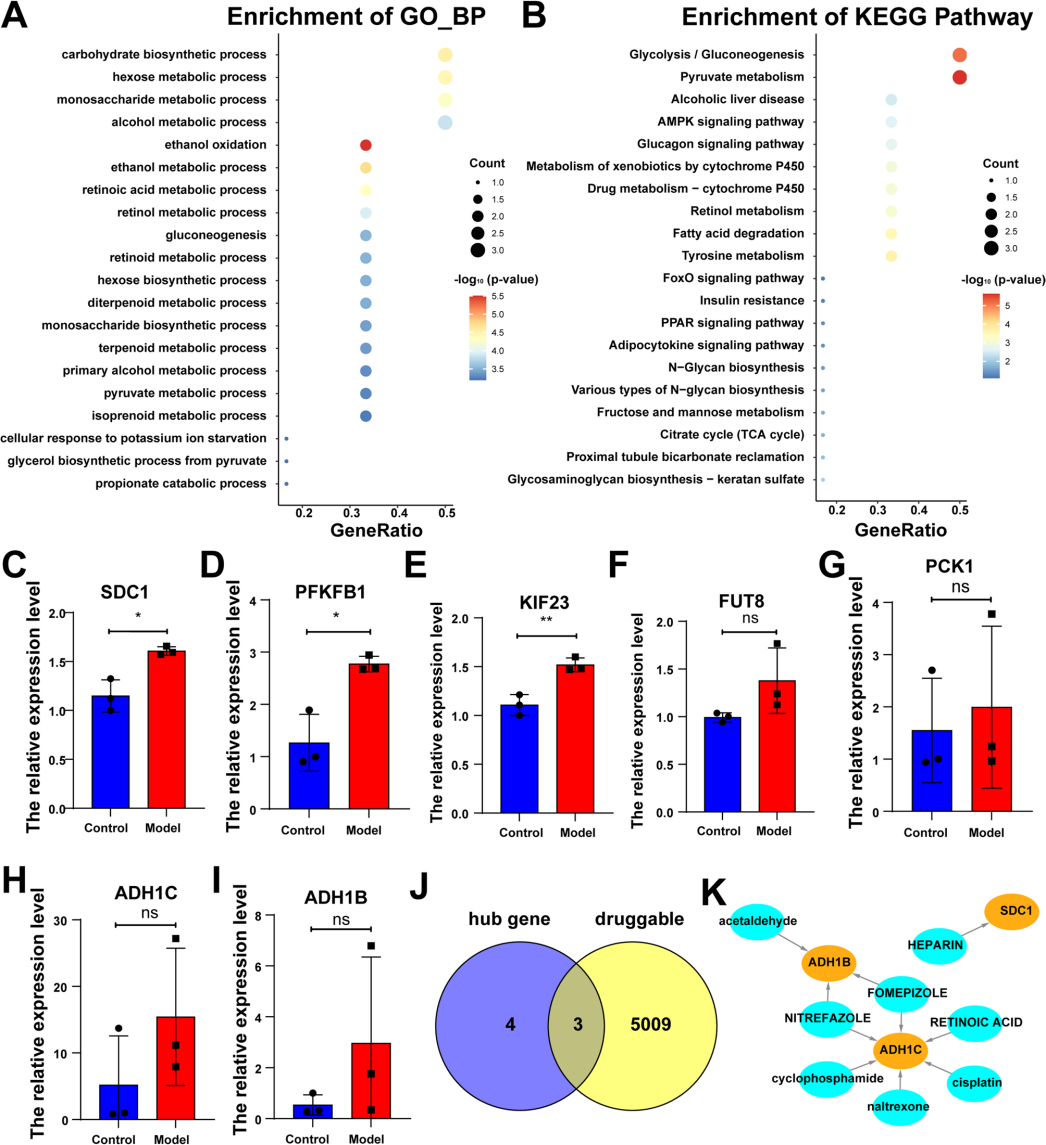
Hub genes LRGs are linked to metabolic reprogramming and exhibit potential druggability. (**A**) GO enrichment analysis of seven hub genes. (**B**) KEGG pathway analysis of seven hub genes. The relative expression level of (**C**) *Sdc1*, (**D**) *Pfkfb1*, (**E**) *Kif23*, (**F**) *Fut8*, (**G**) *Pck1*, (**H**) *Adh1c*, and (**I**) *Adh1b* were validated by qRT-PCR in the control and RA model group. **p < 0.01, *p < 0.05, ns means no significant. (**J**) Venn diagram showing the overlap between the seven hub genes and druggable genes annotated in the DGIdb database. (**K**) Network diagram illustrates the interactions between the druggable hub genes (*Sdc1*, *Adh1b*, and *Adh1c*) and their associated compounds curated in DGIdb

To further elucidate the functional implications of the identified LRGs in RA, we performed enrichment analysis. GO enrichment analysis revealed that seven hub genes were significantly enriched in glucose metabolism-related biological process, including carbohydrate biosynthetic process, hexose biosynthetic process, monosaccharide biosynthetic process, and gluconeogenesis (Fig. 7A). KEGG enrichment analysis further confirmed that the hub genes were significantly involved in multiple metabolic pathways, such as glycolysis/gluconeogenesis, pyruvate metabolism, the TCA cycle, and fructose and mannose metabolism (Fig. 7B). These findings indicate that seven hub genes may participate in metabolic reprogramming in RA. qRT-PCR analysis was conducted in a RA cell model using MH7A cells. The results showed that all seven hub genes exhibited an upregulated trend compared with controls, with SDC1, PFKFB1, and KIF23 reaching statistical significance (*p* < 0.05, Fig. 7C-I). To identify the therapeutic potential of the seven hub genes in RA, DGIdb-based draggability analysis was performed. The results identified three hub genes (*Sdc1*, *Adh1b*, and *Adh1c*) as overlapping with known druggable targets in RA (Fig. 7J). Network analysis further indicated potential interactions between *Sdc1* and HEPARIN, *Adh1b* with acetaldehyde, NITREFAZOLE, and FOMEPIZOLE, and *Adh1c* with RETINOIC ACID, cyclophosphamide, cisplatin, NITREFAZOLE, FOMEPIZOLE, and naltrexone (Fig. 7K). Collectively, these findings indicate that a subset of seven LRGs shows preliminary druggability signals.

## Discusses

RA causes irreversible synovial damage and remains therapeutically challenging [13, 14]. The significance of lactylation modification of proteins in the pathogenesis of autoimmune diseases has been emphasized in recent studies [15]. The pathophysiological involvement of LRGs in RA remains incompletely elucidated. We identified seven key LRGs through integrative bioinformatic approaches, confirmed their aberrant expression, and revealed strong associations with immune cell infiltration patterns in RA. These findings provide new mechanistic insights and suggest potential prognostic indicators and therapeutic targets in RA.

In this study, based on LRGs, we performed the immune infiltration analysis between two subtypes, the CIBERSORT results showed that memory B cells, CD4 naïve T cells, CD4 memory activated T cells and M0 Macrophage were more infiltrated in subtype B. GSVA analysis revealed a significant enrichment of LRGs in the P53 signaling pathway, natural killer cell mediated cytotoxicity, toll like receptor signaling pathway and primary immunodeficiency in RA. The P53 signaling pathway is activated by various stressors, resulting in cell cycle arrest, apoptosis, and cellular senescence [16]. Natural killer cell mediated cytotoxicity is a crucial immune response for countering viral infections and malignancies. Toll like receptor signaling pathway plays crucial roles in the innate immune system [17]. Primary immunodeficiency indicates a higher susceptibility to autoimmune diseases [18]. The above findings indicate that LRGs impact the immune response of RA by acting in the cell cycle and autophagy.

Based on three machine learning algorithms, we identified seven diagnostic feature biomarkers (*Sdc1*, *Pfkfb1*, *Fut8*, *Adh1b*, *Adh1c*, *Kif23* and *Pck1*) with significant diagnostic value. The protein encoded by syndecan-1 (SDC1) is a transmembrane heparan sulfate proteoglycan that regulates key cellular processes, including proliferation, migration, and interaction with the extracellular matrix [19, 20]. SDC1 has been identified as a lactylation-related signature gene in breast cancer [21]. Notably, Emerging studies have demonstrated that metabolic reprogramming of syntenin-1 governs inflammation and angiogenesis in FLS and endothelial cells in RA. This indicates that SDC1 may be involved in immune regulation in RA [22]. In addition, SDC1 with various inhibitors can hinder cell proliferation and migration [23]. Our immune infiltration analyses further revealed that Sdc1 expression were positively correlated with the infiltration abundance of memory B cells and M1/M2 macrophages and localized predominantly to plasma cell clusters of B cells. The *Pfkfb1* gene product is part of the 6-phosphofructo-2-kinase/fructose-2,6-bisphosphatase enzyme family, which has the capability to modulate the glycolytic pathway [24]. Accumulating evidence indicates that *Pfkfb1* participates in the metabolic reprogramming of synovial macrophages under inflammatory conditions in RA [25]. Moreover, B cells are central to humoral immunity and macrophage subsets are key drivers of inflammatory regulation [26, 27]. This suggests that LRGs may serve as a lactylation-modulated regulator of immune dysregulation in RA, linking inflammatory responses with tissue remodeling and disease progression.

Gluconeogenesis (*Pck1*), alcohol dehydrogenase family (*Adh1b*/*Adh1c*) and 6-phosphofructo-2-kinase/fructose-2,6-bisphosphatase enzyme family (*Pfkfb1*) converge functionally on glucose metabolism, a pathway intimately linked to lactate production and lactylation [24, 28, 29]. PCK1 is the main control point for the regulation of gluconeogenesis and can replenish glycolytic reserves in cancer cells, thereby facilitating truncated gluconeogenesis. Given that lactate accumulation is a prerequisite for histone and protein lactylation, aberrant regulation of PCK1 may indirectly modulate lactylation-driven signaling in RA synovial cells. *Adh1b*/*Adh1c* belongs to the alcohol dehydrogenase family that shows high activity for ethanol oxidation and plays an essential role in ethanol catabolism [29]. Study revealed that ADH1B is a new candidate for a mesenchymal tumor suppressor involved in the ADH1B/retinoid-mediated regulation of tumor-promoting IL-6 [30]. Because ethanol oxidation is closely tied to NAD+/NADH balance, altered AHD1B/ADH1C activity may also affect glycolytic flux and lactate generation, thereby influencing lactylation-dependent immune reprogramming in RA [31, 32]. Similarly, *Pfkfb1* controls the levels of fructose-2,6-bisphosphate, a potent allosteric activator of glycolysis. Conclusively, these findings suggest that LRGs may contribute to altered lactate production and lactylation signaling in RA, thereby linking metabolic reprogramming to inflammatory pathogenesis in RA.

*Fut8* encodes a fucosyltransferase that modulates core fucosylation and has been implicated in regulating immune responses in autoimmune diseases [33]. In preclinical models of colitis and in patients with inflammatory bowel disease (IBD), increased FUT8-mediated fucosylation on T cells promotes immune activation, whereas Fut8 deficiency confers resistance by suppressing TCR signaling [34]. Importantly, genome-wide association studies (GWAS) have linked FUT8, as a gene encoding a glycosyltransferase, to autoimmune and inflammatory diseases, including RA [35]. These findings emphasize the potential role of lactylation in RA pathogenesis and offer a foundation for future studies to explore the potential of these lactylation-related genes as prognostic indicators and therapeutic targets.

Our functional enrichment analysis demonstrated that the seven hub LRGs were significantly enriched in glucose metabolism-related pathways. These findings suggest that metabolic reprogramming may be a key mechanism linking lactylation to immune dysregulation in RA, as lactate produced from glycolysis serves as a substrate for lysine lactylation, thereby potentially modulating the expression and function of immune-related genes [36, 37]. Moreover, our results suggest that three hub genes (*Sdc1*, *Adh1b*, and *Adh1c*) may serve as candidate targets for therapeutic intervention in RA. Notably, among these, cyclophosphamide is a clinically approved immunosuppressant for RA [38], and retinoic acid participates in metabolic and immune regulation [39]. Collectively, these findings indicate that hub LRGs may function as key modulators at the interface of metabolic reprogramming and immune regulation in RA, offering both mechanistic insights and potential therapeutic opportunities.

This study has several limitations. First, the analysis was based on publicly available transcriptomic datasets and RA cellular models, which cannot fully recapitulate the systemic and synovial complexity of RA. Second, the absence of non-RA arthritis controls and longitudinal radiographic data limits the ability to establish the diagnostic or prognostic utility of the identified LRGs. Third, while correlations between LRGs and immune infiltration were observed, there is no direct evidence that lactylation itself drives these associations. Finally, these findings just provide new mechanistic insights and potential targets for future validation in larger clinical cohorts.

## Conclusion

In conclusion, our study employing machine learning techniques has pinpointed *Sdc1*, *Pfkfb1*, *Fut8*, *Adh1b*, *Adh1c*, *Kif23* and *Pck1* as distinctive biomarkers for diagnosing RA, linked to the immune system’s infiltration patterns. These discoveries offer novel insights into the progression and candidate therapeutic for RA.

## Supplementary Information

Below is the link to the electronic supplementary material.

Supplementary Material 1

Supplementary Material 2

Supplementary Material 3

Supplementary Material 4

## Data Availability

The datasets used and analyzed during the current study are available from the publicly available GEO database: GSE89408, GSE12021, GSE55235 and GSE55457. There are no ethical issues or other conflicts of interest. The expression data supporting this study are from previously reported databases, which have been cited. The data that support the findings of this study are available anonymously from the corresponding author. Further inquiries can be directed at the corresponding author.

## Abbreviations

RA: Rheumatoid arthritis
LRGs: Lactylation-related genes
LASSO: Least Absolute Shrinkage and Selection Operator
RF-REF: Random Forest Recursive Feature Elimination
SVM: Support Vector Machine
SLE: Systemic lupus erythematosus
RBC: Red blood cell
UPS: Ubiquitin proteasome system
FLS: Fibroblast-like synoviocytes
ART: Antimalarial drug artemisinin
MSiDB: Molecular signatures database
DEGs: Differentially expressed genes
ssGSEA: Single sample gene-set enrichment analysis
GSVA: Gene-set variation analysis
WGCNA: Weighted gene co-expression network analysis
TOM: Topological overlap matrix
MM: Module membership
GS: Gene significance
GO: Gene ontology
KEGG: Kyoto encyclopedia of genes and genomes
DGIdb: Drug-gene interaction database
DMEM: Dulbecco’s modified eagle medium
FBS: Fetal bovine serum
qRT-PCR: Quantitative real-time PCR
SDC1: Synadecan-1
IBD: Inflammatory Bowel Disease
CMFs: Colonic mucosa fibroblasts
ROCK: Rho-associated protein kinase
PDAC: Pancreatic ductal adenocarcinoma
