# Supplementary figures and images for: Identification and validation of lactylation-related genes signature and immune infiltration landscape of rheumatoid arthritis based on machine learning
Source: Hereditas. 2026 May 14;163:81. doi: 10.1186/s41065-025-00579-1 (PMC13343758; doi:10.1186/s41065-025-00579-1)

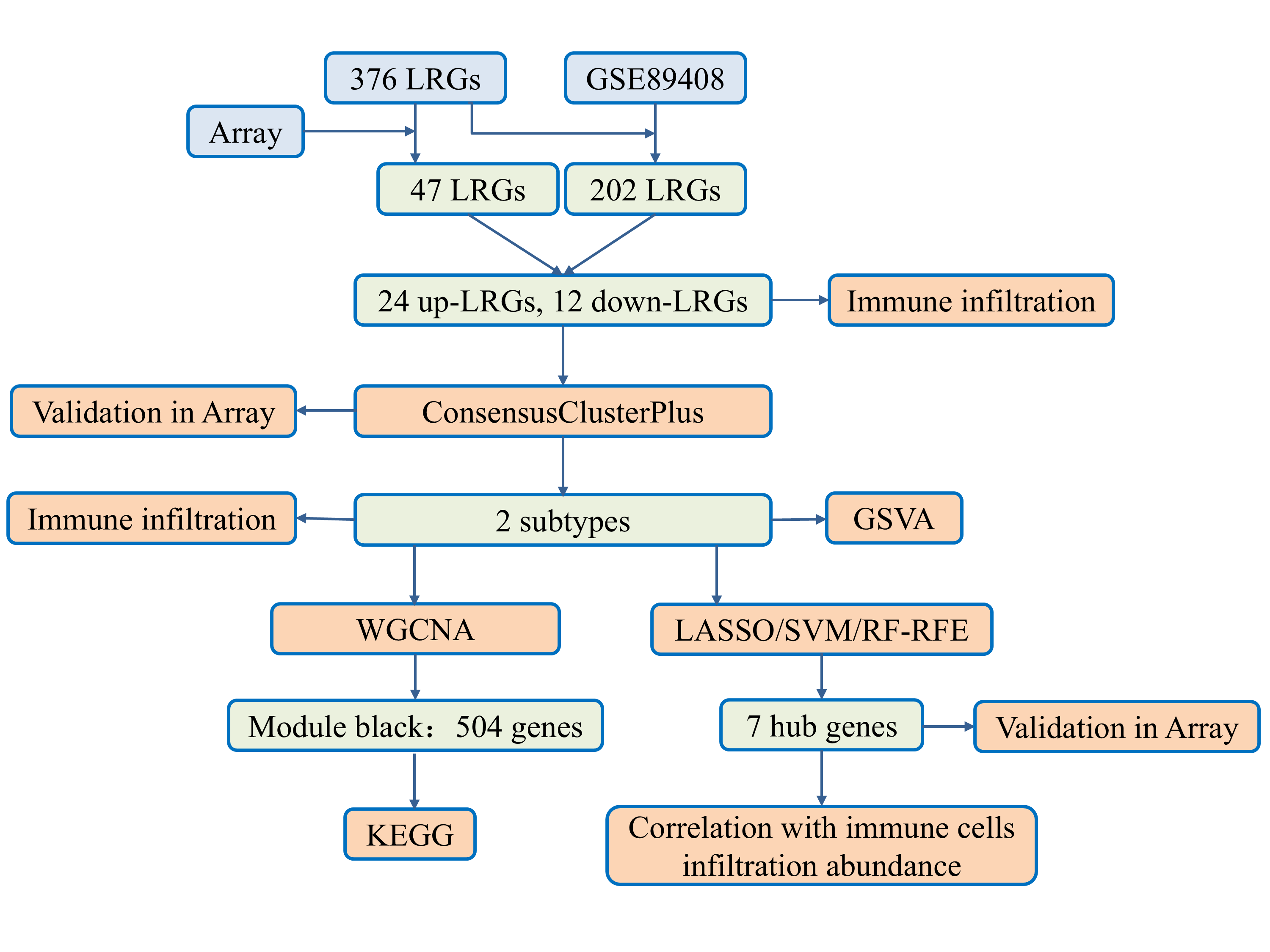

Supplement: Supplementary file 1 — Supplementary Material 1 [file 41065_2025_579_MOESM1_ESM.tif]

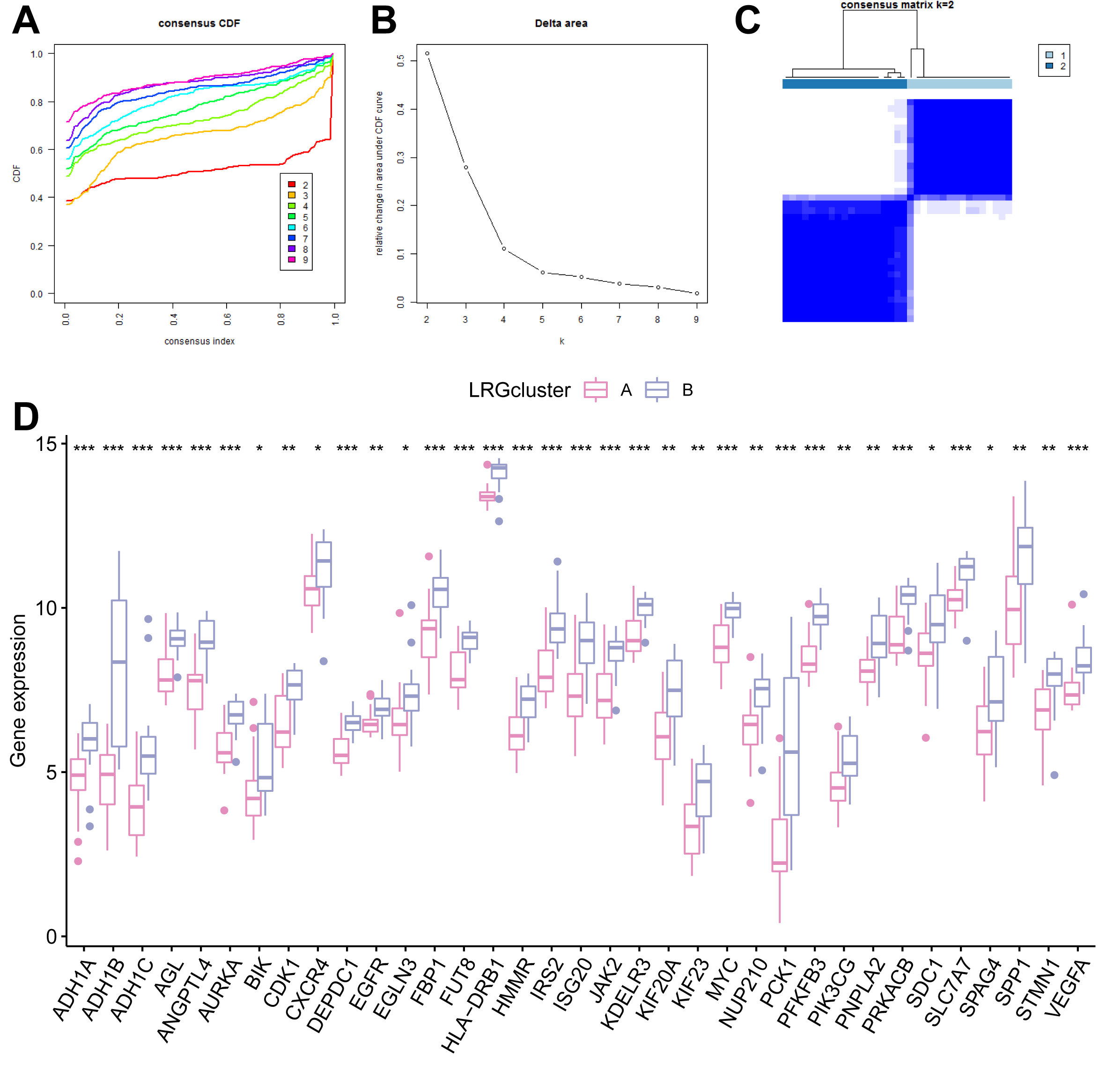

Supplement: Supplementary file 2 — Supplementary Material 2 [file 41065_2025_579_MOESM2_ESM.tif]

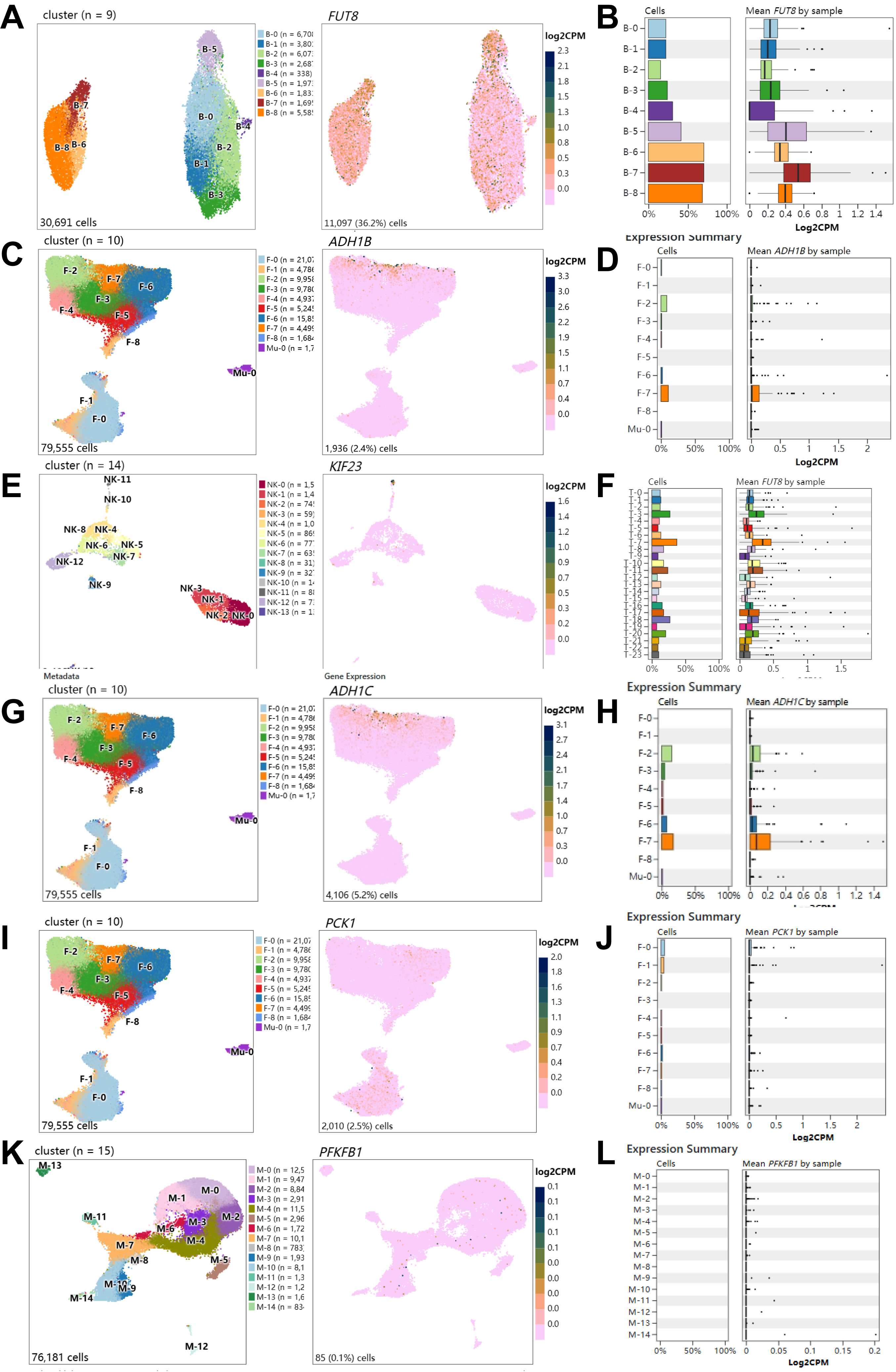

Supplement: Supplementary file 3 — Supplementary Material 3 [file 41065_2025_579_MOESM3_ESM.tif]
